# Social Threat and Motor Resonance: When a Menacing Outgroup Delays Motor Response

**DOI:** 10.3389/fpsyg.2016.01697

**Published:** 2016-11-01

**Authors:** Roberta Capellini, Simona Sacchi, Paola Ricciardelli, Rossana Actis-Grosso

**Affiliations:** ^1^Department of Psychology, University of Milano-BicoccaMilan, Italy; ^2^Milan Center for NeuroscienceMilan, Italy

**Keywords:** social threat, motor resonance, action observation, intergroup relations, MouseTracker

## Abstract

Motor resonance (MR) involves the activation of matching motor representations while observing others’ actions. Recent research has shown that such a phenomenon is likely to be influenced by higher order variables such as social factors (e.g., ethnic group membership). The present study investigates whether and how the perception of a social threat elicited by an outgroup member and by contextual cues can modulate motor responses while an individual observes others’ movements. In an experimental study based on an action observation paradigm, we asked participants to provide answers through computer mouse movements (MouseTracker). We manipulated the agents’ group membership (ingroup vs. outgroup) and the social valence of the objects present in a context (neutral vs. threatening) to elicit social menace through contextual cues. Response times and computer mouse trajectories were recorded. The results show a higher level of MR (i.e., participants started to respond earlier and were faster at responding) when observing an action performed by the ingroup members rather than by the outgroup members only when threatening objects are present in a given context. Participants seem to resonate better with their ingroup; conversely, the outgroup member movements tend to delay motor responses. Therefore, we extend prior research going beyond the general ingroup bias effect on MR and showing that the interaction between membership and contextual cues is likely to elicit threat-related stereotypes. Practical implications of these findings are discussed.

## Introduction

We tend to move with other people around us. For instance, when we see other people dancing or clapping their hands, we spontaneously synchronize our actions with those of our interaction partners (Cappella, 1997; Chartrand and Bargh, 1999). Furthermore, we resonate with others by mentally simulating and mimicking their gestures, postures, facial expressions, and emotions (Decety and Jackson, 2004). The present research focuses on the relationship between others’ action observations and motor resonance (MR) and on the effects of group membership on this phenomenon. More specifically, the present experimental study explores whether and how the perception of social threat elicited by an outgroup member and by contextual cues is likely to modulate motor responses when facing an agent’s action.

Several studies involving the use of different neuroscience techniques ranging from fMRI to TMS (see for instance the body of research on motor neuron systems: Rizzolatti et al., 2001; Rizzolatti and Craighero, 2004; Rizzolatti and Sinigaglia, 2010) and behavioral measures consistently show that observing someone else performing an action elicits a motor activation similar to an activation that occurs when one performing the observed action personally (Fadiga et al., 1995, 2005; Dijksterhuis and Bargh, 2001; Sebanz et al., 2003; Iacoboni, 2005; Fourkas et al., 2006; Press et al., 2011).

Thus, this phenomenon of MR implies one’s capacity to embody a representation of others’ actions, and it seems to contribute to several complex and crucial social skills, such as one’s understanding of others’ actions, intentions and emotions (Hurley, 2008; Iacoboni, 2009) and the facilitation of interpersonal coordination and cooperation (Knoblich and Sebanz, 2006). For this reason, this type of ability is fundamental to our success as individuals and as a species and confers significant adaptive social advantages (e.g., Frith, 2007).

Although MR is an uncontrolled and automatic process (Gallese et al., 1996; Rizzolatti and Craighero, 2004; Wilson and Knoblich, 2005), over the last decade several studies have focused on the possibility that biological, individual and social factors may modulate such an effect.

In this regard, neuroscience evidence (fMRI) suggests that specific motor brain regions (i.e., right rostral parietal foci) are active only when observing biological movements (Perani et al., 2001; see also Kilner et al., 2003) and actions performed by conspecifics (Buccino et al., 2004). Moreover, previous research has shown that tendencies to simulate observed actions (Fadiga et al., 1995, 2005; Urgesi et al., 2006; Aglioti et al., 2008) or sensorimotor states of other individuals (Avenanti et al., 2005; Minio-Paluello et al., 2009) can be affected by individual differences such as gender (Cheng et al., 2008) or high-level personality traits such as empathy (Avenanti et al., 2009).

Among social factors, the actor and perceiver’s group membership seems to play a central role. For instance, Molnar-Szakacs et al. (2007) found that witnessing actions performed by an individual of one’s cultural and ethnic ingroup increases corticospinal excitability to a greater extent than observing actions performed by an outgroup member (see also Liew et al., 2011). In line with this, other recent research has suggested a ‘group bias’ in MR (Gutsell and Inzlicht, 2010, 2013; for exceptions see Désy and Théoret, 2007; Losin et al., 2012). Moreover, such an effect has been proven to be stronger for those presenting high levels of racial prejudice. Starting from the assumption that an ingroup can be conceived of as an extended self (Aron et al., 1992; Brewer and Gardner, 1996), these results are in line with prior findings showing that action observation related regions are more active in response to stimuli associated with the self than with others (Uddin et al., 2006; Kaplan et al., 2008) and when facing agents physically similar to oneself (Molnar-Szakacs et al., 2007).

Although these studies started to explore the influence of social factors on MR, this line of research leaves open questions on the role of social threats in such a process. Social threat has been shown to be crucial to social perception. Indeed, research on impression formation suggests that when evaluating others we are primarily interested in defining whether others could represent an advantage or a threat (Wojciszke et al., 1998; Wojciszke, 2005; Fiske et al., 2007; Cuddy et al., 2008). In addition, perceptions of threat have emerged as an important predictor of global group attitude (Stephan et al., 1999; Stephan and Stephan, 2000; Riek et al., 2006; Pettigrew, 2008; Pettigrew and Tropp, 2008) in early stages of the impression formation process (Todorov et al., 2009). However, not every outgroup is stereotypically associated with social menace in every condition. Specific social categories (e.g., Blacks, Latinos and more recently Arabians) are stereotypically associated with aggression and threat (e.g., Payne, 2001; Mange et al., 2012) and are more likely to elicit aggressive responses in a social perceiver.

Recent literature has explored the role of threatening contextual cues on attention and social perception, showing that evolutionary relevant threatening stimuli are effective at capturing attentional resources (Öhman et al., 2001; Fox et al., 2002) thus causing interference with goal-directed activity (Williams et al., 1988, 1996). People are sensitive to dangerous objects (Anelli et al., 2012a,b, 2013), and the dangerousness of everyday graspable objects can influence one’s surrounding context and the boundaries of peripersonal space (Coello et al., 2012). Furthermore, as revealed by previous studies, contexts systematically influence social categorizations (Freeman et al., 2013), may modify the interpretation of what a facial expression represents (Righart and De Gelder, 2008) and may affect person perception. Hence, contextual cues may influence the impression of a social target. Threatening contextual cues, for instance, can weaken or strengthen race-based stereotypes of aggressiveness and menace (Trawalter et al., 2008): a threatening context is likely to activate negative stereotypes associated with specific social categories.

Building on this body of work, the present study aimed to explore whether and how MR triggered by observations of others’ arm movements toward an object can be modulated by social variables such as ethnic group membership. Specifically, we predicted an increased MR when participants observe an action performed by an ingroup member rather than by an outgroup member in line with prior studies (Gutsell and Inzlicht, 2010). In going beyond prior research showing that MR is modulated by social categorization, we investigated whether the perceived threat posed by a social target is likely to modulate a social perceiver’s MR response. More specifically, we expected that social threat elicited by a specific outgroup (i.e., stereotypically aggressive) in a specific context (i.e., threatening contextual cues) is likely to amplify the pattern. This hypothesis is in line with previous studies on the effect of morality on MR (Liuzza et al., 2015), which shows that the phenomenon is significantly reduced when observing immoral actions (namely actions related to social threats; Brambilla et al., 2013) in individuals presenting high levels of harm avoidance.

To investigate these hypotheses, we carried out an experimental study where using an action observation paradigm we asked participants to provide responses through computer mouse movements. More specifically, participants observed a movie clip showing an actor moving his arm toward an object; then on the screen, a square appeared in a congruent or incongruent position relative to the direction of the actor’s movement. Participants were asked to indicate the square position by performing a computer mouse movement toward one of two labels denoting the position (i.e., left or right). We manipulated the group membership of the actors (ingroup vs. outgroup) and the social valence of objects present in the context (neutral vs. threatening) to elicit social menace through contextual cues. MR has been assessed through the use of an action observation paradigm implemented by MouseTracker software (Freeman and Ambady, 2010), a tool that measures behavioral responses by recording computer mouse trajectories and that provides multiple informative dependent variables as detailed in the results section [e.g., initial response times, total response times, the maximum deviation (MD) point of the trajectory and the area under the curve (AUC)].

Moreover, our paradigm, which orthogonally manipulates directions of an actor’s arm movement and participant’s response direction, allowed us to distinguish the effect of MR from effects elicited by social attention. Indeed, whether the underlying process was social attention, we would expect to find task facilitation when target-stimuli appeared in a position congruent with the direction of the actor’s movement when compared to incongruent positions. Otherwise, if we measured MR, we would expect to find task facilitation when actors move the same arm as the one used by participants to provide their answers. If the congruence between response directions and actors’ arm movements does not have an effect, the hypothesis on social attention may be discarded.

## Materials and Methods

### Participants

The initial sample comprised 82 participants who volunteered to participate in the study in exchange for course credit. Seventy-nine participants were Italian citizens. Three non-Italian participants (1 Ukrainian, 1 Peruvian, and 1 Italo-Argentine) were excluded. Thus, the final sample included 79 participants (*M*_age_ = 23.43, *SD*_age_ = 3.76, range 18–44 years, 40 males, 39 females). Sixty-seven participants were right-handed, 10 were left-handed, and 2 were ambidextrous according to self-reports; all of them were naive as to the purpose of the experiment. An a priori power analysis for within-subject ANOVA (medium effect size = 0.20; power = 0.95) suggested minimum *N* = 46. We advertised the study and enrolled all individuals who had responded and volunteered to participate.

### Ethical Statements

All participants provided written informed consent before participating in the study. The study was conducted in accordance with the ethical standards outlined in the 1964 Declaration of Helsinki and with the standard ethical procedures recommended by the Italian Association of Psychology (AIP). The study was specifically approved by the local Ethics Committee of Milano-Bicocca University.

### Materials and Procedure

The experiment was carried out in a dimly lit room. Participants were asked to complete a questionnaire. On the cover page of the questionnaire, participants provided their demographic data. Participants were then presented with a 7-item national identification scale (e.g., “*I identify* with Italians”; Cameron, 2004), a 9-item Modern Prejudice Scale for Islamic people (e.g., “*For Italians it’s normal to have a relationship with an Islamic person*”; McConahay, 1986), and a 10-item Motivation to Respond Without Prejudice Scale (e.g., “*Being non-prejudiced toward Islamic people is important to my self-concept*”; Plant and Devine, 1998). Participants answered these questions on a 7-point scale ranging from 1 (*not at all*) to 7 (*very much*).

The second part of the experiment was run on an Intel^®^ Pentium^®^ G630 @ 2.70 GHz personal computer interfaced with a 22-in LCD computer monitor (Asus^®^ VW226; Resolution: 1680 pixels × 1050 pixels; Refresh rate: 59 Hz) equipped with the MouseTracker software program (Freeman and Ambady, 2010). After signing the consent form, participants were comfortably seated in a chair positioned approximately 60 cm away from the monitor from which they received instructions and were presented with photos and brief descriptions of the actors they were going to watch during the experiment (name, age, and nationality). Hence, the ingroup (Gabriele, 26 years old, Italian) and outgroup targets (Haashim, 27, Iraqi) were introduced.

The experiment was then conducted. Each trial began with the computer screen showing a small box labeled “Start” at the lower center of the screen and two response boxes labeled “Left” and “Right” on the upper left and upper right corners of the screen, respectively. After 500 ms, a random video (WMV format; 25 frames/s; 640 pixels × 480 pixels; 1.296 kbps; Duration = 1.388 ms) was shown at the center of the screen.

**FIGURE 1:**
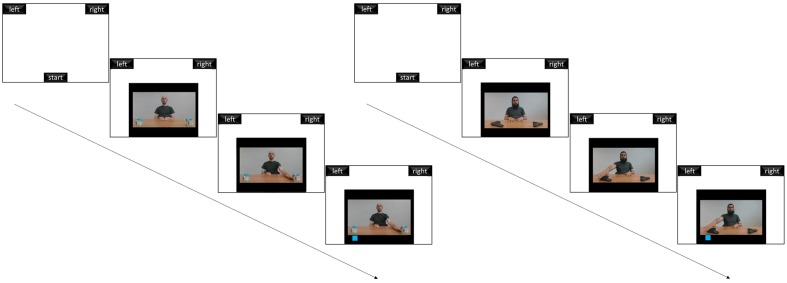
**An example of the experimental procedure**.

As is shown in **Figure 1**, videos presented the front view of an actor performing an arm movement toward one out of two objects located on a table on his left and right, at a distance of 55 cm from his torso and 67.5 cm apart from each other. In all of the videos, the actor looked straight ahead and did not move any body parts other than his arm. The actors used their right hand to move to the right (the participant’s left) and left hand to move to the left (the participant’s right). At the end of each movie clip, a blue square appeared to the left or right of the screen. Participants were required to ignore the direction of the actor’s arm movement and to indicate with a computer mouse movement the position (left or right) in which the square appeared. They were asked to do this as quickly and accurately as possible by moving their computer mouse cursor from the “Start” button to the chosen response box at the top. Responses were allowed only after the square appeared. A blank screen of 500 ms was inserted between each response box click and the following trial.

We manipulated within participants the actor’s ethnic membership (ingroup vs. outgroup) by presenting an Italian and an Arabian target, which is an outgroup stereotypically related to aggression or threat as suggested by recent research (Oswald, 2005; Mange et al., 2012). Moreover, the object valence (neutral vs. threatening) was manipulated by showing box of juice as a neutral object or a gun as a menacing object. In a first pretest, 57 Italian participants (26 males; age range: 18–65; *M*_age_ = 33.20, *SD*_age_ = 10.75) were presented with a picture of Haashim and a picture of Gabriele, and they were asked to evaluate how much they perceived the targets (the order was properly balanced) as threatening (“*[…] is a threatening person”*) and frightening (“*[…] is a frightening person”*) on a 5-point scale (ranging from 1 = *not at all* to 5 = *extremely;*α = 0.76). In line with prior studies (Mange et al., 2012), the results showed that participants perceived the Arabian outgroup member to be more menacing (*M* = 2.26, *SD* = 1.02) than the Italian ingroup member (*M* = 1.96, *SD* = 0.87), *t*(56) = 2.12, *p* = 0.04.

In a second pretest, 14 Italian participants (six males; age range: 21–32; *M*_age_ = 26.07, *SD*_age_ = 3.95) were presented with a picture of a gun and a picture of a box of juice and they were asked to evaluate how much they perceived the object (the order was properly balanced) as threatening (“*[…] is a threatening object”*) and frightening (“*[…] is a frightening object”*), α = 0.86, and graspable (“*[…] is a graspable object”*) on a 5-point scale (ranging from 1 = *not at all* to 5 = *extremely*). Then, they were asked to rate their overall impression on a 7-point scale range (ranging from -3 = *extremely negative* to +3 = *extremely positive*). The results showed that participants perceived the gun to be more menacing (*M* = 4.50, *SD* = 0.71) than the box of juice (*M* = 1.14, *SD* = 0.36), *t*(13) = 18.17, *p* < 0.000, and more negative (*M* = 2.50, *SD* = 1.7) than juices (*M* = 4.93, *SD* = 1.21), *t*(13) = 4.57, *p* < 0.000. Moreover, guns were perceived to be as graspable as the boxes of juice, *p* = 0.90.

We thus showed a total of 16 different videos as a result of combinations of these four variables (membership: ingroup vs. outgroup; actor’s movement direction: left vs. right; object valence: neutral vs. threatening; square position: left vs. right). Each video was presented randomly six times throughout the experiment, resulting in a total of 96 trials with four additional trials presented at the beginning of the session as training trials, producing a total of 100 trials. Trials were split into two blocks: half of the participants were presented with a first block showing actors moving toward neutral objects (e.g., box of juice) followed by a second block with actors moving toward threatening objects (e.g., gun); the other half was presented with a first block showing actors moving toward threatening objects followed by a second block with actors moving toward neutral objects.

Hence, the experimental design consisted of a 2 (block order: neutral first vs. threatening first) × 2 (membership: ingroup vs. outgroup) × 2 (object valence: neutral vs. threatening) × 2 (actor’s movement direction: left vs. right) × 2 (square position: left vs. right) design, with the first factor manipulated between subjects and the latter factors manipulated within subjects. It is important to note that ‘left’ and ‘right’ always refer to the participants’ point of view (the position on the screen); thus, for instance an actor’s movement to left means an actor’s movement performed with his right hand to his right side. Moreover, as we excluded errors from the data, square positioning corresponds to the direction of a participant’s response. Participants were randomly assigned to one of the two experimental groups. As provided by the MouseTracker software, the initial times (IT), response times (RT), AUC, and MD measures were recorded.

## Results

### Preliminary Analysis

Regarding the mouse-tracking data, we conducted the analyses on four different indices provided by MouseTracker that represented our crucial dependent variables. Indices are the following: initial response times (IT), that represents the starting point in which participants begin the mouse trajectory in order to indicate their responses; total RT, which represents the total amount of time taken by participants to perform their responses, MD, which is a common index for assessing response competition, and AUC, which represents another index for evaluate response competition in terms of larger positive AUC values that indicate greater response competition.

Seventy-seven of the 79 participants performed the task using the mouse with their right hand. Since it was not feasible to conduct statistical analyses on two cases, participants who performed the task using their left hand were excluded from the sample. Then, training trials have been eliminated from the analysis. Moreover, trials in which participants did not provide a correct response (e.g., answer ‘left’ when the square compared on the right or vice versa) were discarded (15 of 7,968 recorded trials, corresponding to 0.19%). Next, we removed trials in which the dependent variables indices (IT, RT, AUC, and MD) were greater or lower than ±2.5 SD. With this procedure 485 trails were removed (485 of 7,968 recorded trials, corresponding to 6.09%). Finally, we excluded three outlier data points with standardized values greater than ±3 from the IT, RT, MD, and AUC averages. Thus, final analysis was conducted on a sample of 74 subjects.

Regarding the three explicit scale analysis, after reversing the items negatively phrased in the questionnaire and testing the scales reliability (national identification: Cronbach’s α = 0.72; prejudice: Cronbach’s α = 0.79; motivation to avoid prejudice: Cronbach’s α = 0.85), we computed the average scores for each measure.

Then, to control for possible effects of block order and square position, a 2 (block order: neutral first vs. threatening first) × 2 (membership: ingroup vs. outgroup) × 2 (object valence: neutral vs. threatening) × 2 (actor’s movement direction: left vs. right) × 2 (square position: left vs. right) ANOVA was computed, using the first variable as between-participants factor and the other variables as within-participants factors.

The ANOVA carried out on the crucial dependent variables (IT, Total RT, MD, and AUC) did not reveal any interaction effect with block order (*p*_s_ > 0.05).

Moreover, the five- and four way interaction with square position (corresponding to the participant’s response direction) was non-significant (*p*_s_ > 0.05). This result was crucial in order to exclude a possible influence of social attention (namely the congruence between agent’s movement direction and the position of the target stimulus to which participants were called to respond). Indeed, if the underlying process were due to social attention, we would expect to find task facilitation when square appeared in a position that was congruent with respect to the direction of the actor’s arm movement (e.g., actor’s movement toward left – square on the left) and, on the other hand, to find a greater response delay in incongruent positions (e.g., actor’s movement toward right – square on the left).

Also participants’ handedness, when introduced as factor in the aforementioned analysis, proved to be ineffective (*p*_s_ > 0.05).

Therefore, we collapsed data across these factors and the following analyses do not consider these variables.

### Initial Times

After the preliminary analyses a 2 (membership: ingroup vs. outgroup) × 2 (object valence: neutral vs. threatening) × 2 (actor’s movement direction: left vs. right) within participants ANOVA on the IT was computed.

In line with the hypothesis, the analysis yielded a significant main effect of membership, *F*(1,73) = 17.41, *p* < 0.001, $\eta_{p}^{2}$ = 0.19; indeed, participants started their response with mouse earlier when observing an ingroup actor arm’s movement (*M* = 78.99, *SD* = 3.08) than when observing an outgroup actor (*M* = 85.89, *SD* = 3.31). This result can be interpreted as a higher level of motor activation when perceiving ingroup members’ acts.

**FIGURE 2:**
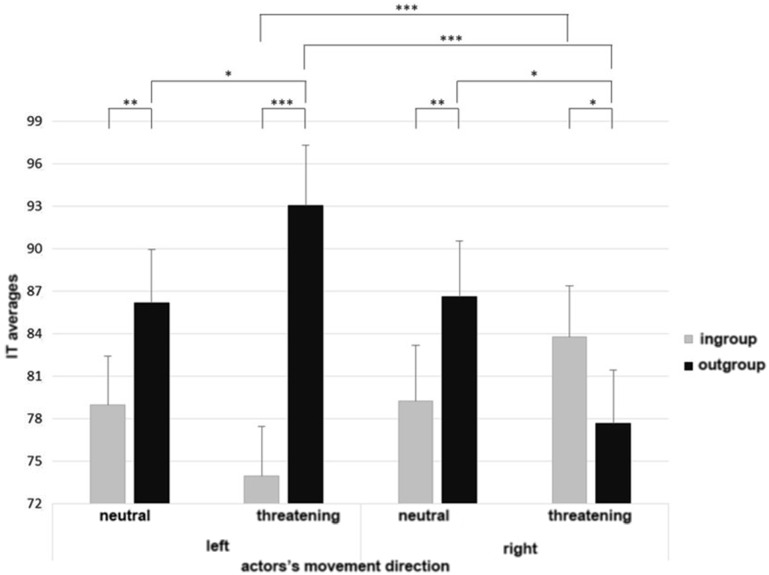
**Three-way interactions between membership, actor’s movement direction, and object valence [Initial Times (IT)].** Asterisks highlight significantly different means comparisons (^∗^*p* < 0.05, ^∗∗^*p* < 0.01, ^∗∗∗^*p* < 0.001). Error bars represent standard errors.

As displayed in **Figure 2**, the ANOVA yielded a significant three-way interaction between membership, actor’s movement direction, and object valence, *F*(1,73) = 21.89, *p* < 0.001, $\eta_{p}^{2}$ = 0.23. As showed by *post hoc* analyses (LSD tests), when the objects presented in the context were threatening (i.e., guns), and the actor was an ingroup member, participants were faster while perceiving the agent moving to left (it is worthy to note that this condition would imply MR since the actor in front view was executing the movement with his right hand, that was the same hand participants were using to perform the task; *M* = 73.95, *SD* = 3.49) than to the right (*M* = 83.80, *SD* = 3.57), *p* < 0.001. On the other side, with threatening object but in outgroup condition, participants were even slower while perceiving the agent moving to the left (*M* = 93.04, *SD* = 4.27) than to the right (*M* = 77.70, *SD* = 3.76), *p* < 0.001. These results can be interpreted as a higher MR with the ingroup member; in stark contrast, the outgroup member’s movement seems to delay motor response. Interestingly, in the other block, when the objects were neutral, in ingroup condition, there was no difference in IT between trials directed to the left (*M* = 78.98, *SD* = 3.42) and to the right (*M* = 79.24, *SD* = 3.93), *p* = 0.94. Analogously, in outgroup condition, no difference arose in IT between trials directed to the left (*M* = 86.17, *SD* = 3.80) and to the right (*M* = 86.64, *SD* = 3.93), *p* = 0.86. Thus, these results seem to suggest that the crucial interaction between actor’s movement direction and membership arouses only when cues in the social context elicit threat.

Moreover, *post hoc* analyses (LSD tests) showed also significant differences in IT between neutral and threatening objects when participants observed an outgroup member; in particular, they were faster when the actor was moving to the left toward the box of juice than when he was moving to the left toward the gun, *p* = 0.05. Conversely, they were slower when the outgroup member was moving to the right toward the box of juice than when he was moving to the right toward the gun, *p* = 0.03. Interestingly, no differences arose in IT between neutral and threatening objects in the ingroup condition, neither when the actor was moving to the left, *p* = 0.14, nor to the right, *p* = 0.16.

Moreover, the ANOVA revealed a significant two-way interaction between membership and actor’s movement direction, *F*(1,73) = 17.83, *p* < 0.001, $\eta_{p}^{2}$ = 0.20, which may be justified by the three-way interaction. The analysis did not yield any other significant effect, *p*_s_ > 0.36.

### Total Response Times

Then a 2 (membership: ingroup vs. outgroup) × 2 (object valence: neutral vs. threatening) × 2 (actor’s movement direction: left vs. right) ANOVA on total RT was carried out.

**FIGURE 3:**
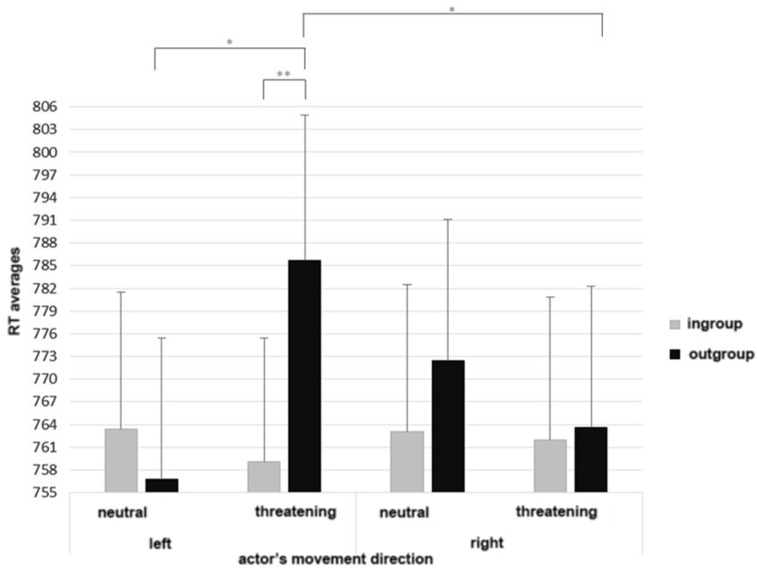
**Three-way interactions between membership, actor’s movement direction, and object valence (Total Response Times).** Asterisks highlight significantly different means comparisons (^∗^*p* < 0.05, ^∗∗^*p* < 0.01, ^∗∗∗^*p* < 0.001). Error bars represent standard errors.

As displayed in **Figure 3**, the ANOVA yielded a significant three-way interaction between membership, actor’s movement direction, and object valence, *F*(1,73) = 5.84, *p* = 0.02, $\eta_{p}^{2}$ = 0.07.

As showed by *post hoc* analyses (LSD tests), when the objects presented in the context were threatening and the actor was an outgroup member, participants were slower when observing an agent moving to the left (*M* = 785.73, *SD* = 19.19) than to the right (*M* = 763.64, *SD* = 18.59), *p* = 0.03. On the other side, in ingroup condition with menacing objects, no differences in RT were revealed between trials directed to the left (*M* = 759.09, *SD* = 16.30) and to the right (*M* = 761.97, *SD* = 18.84), *p* = 0.75.

Interestingly, in the other block, when the objects were neutral and in outgroup condition, participants were faster when the agent was moving to the left (*M* = 756.75, *SD* = 18.72) than to the right (*M* = 772.49, *SD* = 18.62), *p* = 0.08. In ingroup condition with neutral objects, no differences in RT were revealed between trials directed to the left (*M* = 763.71, *SD* = 18.15) and to the right (*M* = 763.11, *SD* = 19.36), *p* = 0.97.

*Post hoc* analyses (LSD tests) showed also significant differences in IT between neutral and threatening objects when the agent was an outgroup member; in particular, when he was moving to the left, participants were slower in the threatening condition (i.e., with guns) than in the neutral one (i.e., with box of juice), *p* = 0.04. There were no differences between neutral and threatening objects when the outgroup member was moving to the right, *p* = 0.59. No differences in RT arose between neutral and threatening objects in ingroup condition, neither when the agent was moving to the left, *p* = 0.70, nor to the right, *p* = 0.93. Furthermore, the ANOVA revealed a significant two-way interaction between object valence and actor’s movement direction, *F*(1,73) = 3.94, *p* = 0.05, $\eta_{p}^{2}$ = 0.05, which may be justified by the three-way interaction. In sum the results partially confirmed data on IT. We should consider that the present index represents the total time used by participants for providing their response, thus participants could use the entire time of the trajectory to adjust their answer, until they clicked the response box.

No other effects were found, *p*_s_ > 0.17.

### Maximum Deviation and Area Under the Curve

We also computed a 2 (membership: ingroup vs. outgroup) × 2 (object valence: neutral vs. threatening) × 2 (actor’s movement direction: left vs. right) ANOVA on two other indices used to measure the response competition: MD and AUC. As shown, using MD versus AUC for the same data does not substantially change the results (Freeman et al., 2008). No effects were found neither on MD nor on AUC, *p*_s_ > 0.08.

These results revealed that participants did not experience response competition in providing their mouse responses. The fact that we found effects on initial and RT and not on the trajectories can be ascribed to the type of task. As shown, we asked participants to indicate the position (left/right) in which the stimulus appeared, that is a simple and quite effortless task. The easiness of this kind of task could have led participants to provide their responses without uncertainties.

### Explicit Scales

Finally, in order to investigate whether participants’ level of identification with the national group (i.e., Italians), level of prejudice and level of motivation to avoid prejudice were likely to moderate the effects, moderated moderation models were explored using PROCESS macro (Hayes, 2013; model 3, 5000 bootstrap resampling) with actor’s movement direction (left vs. right; left defined the condition in which participants provided their responses using the same hand as the one used by the actor in the videos) as an independent variable, membership (ingroup vs. outgroup) as a moderator, the explicit measures as moderator of the moderator, and IT and total RT as dependent variables. The explicit measures used were: identification with the ingroup (*M* = 4.16, *SD* = 0.91), prejudice toward Islamic people (*M* = 3.20, *SD* = 0.87), and motivation to avoid prejudice (*M* = 3.88, *SD* = 1.06). None of these models revealed significant interaction, *p*_s_ > 0.35. Thus, these measures of identification and prejudice did not moderate our effects on MR revealed by previous analyses.

In line with our hypothesis, the results suggest that people are prone to a higher level of MR when observing an action performed by the ingroup members rather than by the outgroup members. Hence, participants seem to resonate better with their ingroup; conversely, outgroup members’ movements tend to delay motor responses. Moreover, the perceived social threat elicited by socially menacing cues interferes with this effect; when participants observed an outgroup member moving toward a weapon, they were slower at providing responses. Thus, our results seem to suggest that the crucial interaction between actor’s movement direction and membership occurs only when cues in a social context elicit threat.

## Discussion

A robust line of research has widely suggested that observing another person’s action activates corresponding motor representations in the observer (Iacoboni et al., 1999; Rizzolatti et al., 2001; Rizzolatti and Craighero, 2004); moreover, prior studies have shown that MR may be influenced by characteristics of an action made and of the performer of that action (Molnar-Szakacs et al., 2007; Cheng et al., 2008). The present study investigated whether and how perceptions of social threat elicited by an outgroup member and by social cues can modulate motor responses when a person observes others’ actions.

Our findings suggest that MR during action observation is likely to be modulated by ethnic group membership. Indeed, consistent with our hypothesis and in line with the existing literature (Molnar-Szakacs et al., 2007; Avenanti et al., 2010; Gutsell and Inzlicht, 2010, 2013), participants tended to resonate better with their ingroup; in fact, when the ingroup actor executed a movement with the same hand as the one used by participants to provide their responses, motor facilitation was found. Conversely, when the outgroup member performed an action using the same hand as participants, a delay in response was found.

These results can be interpreted in the light of the existing literature on group membership and social interactions. Indeed, people empathize more with the ingroup members than the outgroup members (Dovidio and Gaertner, 2010; Trawalter et al., 2012; Gutsell and Inzlicht, 2013) and tend to perceive the ingroup members more favorably and as more similar to themselves (Hewstone et al., 2002). Studies on spontaneous synchrony, mimicry and motor coordination consistently show that individuals are less likely to synchronize their movements with those whom they harbor negative feelings for (Miles et al., 2010). Moreover, this effect may be partially due to familiarity: ingroup members are usually the ones with whom we most often interact (Fiske, 1992). Therefore, moving with others or resonating with their movements could be considered an early embodied form of relation with our conspecifics that could be affected by social perception and cultural inter-individual differences (Sacheli et al., 2015).

However, the present research complements and extends this emerging line of research, going beyond a mere ingroup bias effect and exploring the role of social threat. Our main finding suggests that the perception of social threat, elicited by an outgroup member stereotypically associated with social aggression (Oswald, 2005) and by contextual cues, is likely to interfere with motor response provided when facing an agent’s action. In fact, when participants faced a stereotypically aggressive outgroup member moving toward a weapon, they were slower to initiate motor responses. Interestingly, the interaction effect between membership and movement direction disappeared when contextual cues were neutral and unlikely to elicit social threat.

It is worthy to note two additional nuances of our results. First, in neutral conditions, the MR effect disappeared when participants viewed an ingroup member. One possibility for why we did not find MR in this condition is because in this specific context (i.e., when threat is not salient), an individual may be likely to focus on the task and to disregard social stimuli. By contrast, when social threat is elicited, an individual is more heavily influenced by the presence and movements of social targets that can become potentially menacing. As highlighted by the literature of threat and attention, evolutionary relevant threatening stimuli are effective at ensnaring attentional resources (Öhman et al., 2001; Fox et al., 2002), thus interfering with goal-directed activity (Williams et al., 1988, 1996).

Second, no difference was found between neutral and threatening conditions when participants faced the ingroup members: RT differed between neutral and threatening conditions only when participants observed an outgroup agent’s movements. This pattern could suggest that threatening objects are likely to activate negative stereotypes associated with specific social categories (e.g., Arabians). As revealed by a prior study, threat cues might weaken or strengthen race-based stereotypes of aggressiveness and menace (Trawalter et al., 2008): hence, only in particular conditions (e.g., in our experiment when participants were presented with guns), negative stereotypes associated with racial minorities may be active and likely to garner attention. As argued by Trawalter et al. (2008), information gleaned from bottom-up (e.g., contextual cues) and top-down processing (e.g., stereotypic expectancies) may have interactive effects on social perception. From these findings, future studies could further explore effects of group membership by presenting participants with control outgroups not stereotypically associated with threat or aggression. In this way, it would be possible to control whether effects that emerge are to be ascribed to the presence of a generic outgroup or to a particular and specific menacing outgroup.

This result seems to be at odds with previous research (Gutsell and Inzlicht, 2013) showing that when outgroup behavior is negative and threatening, individuals begin to process them as ingroup members, thus reducing ingroup bias effects on MR. However, the results are in line with functional perspectives on person perception (e.g., Todorov et al., 2009), suggesting that when an outgroup behavior is threatening, people can allocate cognitive resources to the threatening individual. This form of cognitive resource allocation does not necessarily apply to active movements. In fact, the delay found in response to the Arabian outgroup member as he moved toward a gun can be interpreted as a freeze reaction to a harmfully perceived event as suggested by several studies that show that spontaneous body responses to social threat cues elicit freeze-like behaviors in humans (Roelofs et al., 2010). Moreover, our findings are in line with previous studies on the effects of morality on MR (Liuzza et al., 2015) showing a decrease in MR when observing immoral actions, in particular in individuals presenting high levels of harm avoidance. As an extension of this work, it will be interesting to investigate the relation between MR and visual attention. Indeed, future works could explore whether motor freezing arising as a reaction to a menacing outgroup could be associated with greater visual attention to this agent; participants, for instance, could be attracted to a menacing target while at the same time being frozen in their motor reactions. For this reason, it will be useful to integrate the measure of RT and computer mouse trajectories provided through MouseTracker with the analysis of eye movements using an eye tracker.

Furthermore, an added value of this work lies in the experimental methodology we adopted; on one hand, we used an action observation paradigm implemented using MouseTracker software (Freeman and Ambady, 2010), which is a tool that measures behavioral responses by recording computer mouse trajectories and that provides multiple informative dependent variables (e.g., initial RT, total RT, MD point of the trajectory, and AUC). In this way, it is possible to monitor and implicitly investigate the entire motor response to understand at what level of the process the effects interfere. Thanks to this experimental tool, it was possible to highlight that the effects of our variables are particularly precocious, as they interfered with the task early on in the process (IT).

Moreover, the paradigm we used allows to discriminate between two competing underlying processes. Through our action observation paradigm, we presented participants with congruent (i.e., actor’s movement toward the left – square on the left) or incongruent (i.e., actor’s movement toward the right – square on the left) stimuli. Several studies on social attention that have adopted a modified version of the Posner paradigm (Posner, 1978, 1980; Frischen et al., 2007) and the Social Simon Paradigm (Tsai and Brass, 2007; Dolk et al., 2011) manipulated the congruence of stimuli in the same way. In our study, the orthogonal manipulation of the square’s position (corresponding with participant response directions) alongside membership and actor’s movement directions allowed us to disentangle effects of MR from effects elicited by social attention. Indeed, if the underlying process were due to social attention, we would expect to find task facilitation when stimuli (i.e., square) appeared in a position congruent to the direction of the actor’s movement. With incongruent positions, we would expect to find greater response competition and thus an increase in the difficulty in making a decision. Otherwise, if we measured MR, we would expect to find task facilitation with an actor moving the same arm as the one used by participants to provide their answers. The motor facilitation result found when the actors moved the same arm as the one used by participants to provide their answers supports the hypothesis on MR. Moreover, as the square’s position did not interact with our crucial effects, the hypothesis on social attention can be discarded.

Moreover, we showed that our findings are not affected by levels of prejudice; both people with a high level of prejudice toward Arabian people and those with low prejudice were influenced when performing the task. From a social psychology perspective, the results are compatible with stereotype activation rather than with prejudice effects. Stereotypes are fixed and over-generalized beliefs about people that are based on their membership to a particular social category (for a review, see Hilton and von Hippel, 1996). Previous research has widely demonstrated that trait concepts and stereotypes, which are generally shared within a community and stable over time, become automatically active in the presence of a relevant behavior or stereotyped-group features. Given their pervasiveness in cultural contexts, stereotypes can have detrimental effects on social perception and elicit stereotype-consistent behavioral responses independent from the individual’s attitudes and personal values (i.e., prejudices) toward that social group (Devine, 1989; Devine and Elliot, 1995). From our results and in consideration of the fact that most people internalize stereotypes in the course of normal socialization, future studies could also investigate the role of individual characteristics (e.g., age) in the development of stereotypes. From a lifespan perspective, it will be interesting to explore the development of adaptive human skills (Knoblich and Sebanz, 2006) to resonate with co-specifics during different stages of human life and particularly in relation to ingroup bias and social threat perception.

Finally, from an intergroup point of view, the effect of social threat on MR could have important implications; such an effect may affect the first stage of social perception and may alter communication and interactions with outgroup individuals and the quality of intergroup relations. Moving together and coordinating with other movements can bridge the gap between the self and others and create a sense of social connection. Therefore, investigating the effects of facilitating and hindering factors of MR could help understanding the challenges associated with intergroup interactions. To conclude, the present study provides avenues for further studies on the role of variables likely to reduce intergroup threats and to promote more cooperative relations between members of different social groups.

## Author Contributions

RC drafted the first version of the manuscript. RC and SS run the study and conducted data analyses. RC, SS, PR and RA-G conceived the study idea and the experimental paradigm and provided comments on the first version of the manuscript.

## Conflict of Interest Statement

The authors declare that the research was conducted in the absence of any commercial or financial relationships that could be construed as a potential conflict of interest.

The reviewer FA and handling Editor declared their shared affiliation, and the handling Editor states that the process nevertheless met the standards of a fair and objective review.

## References

[B1] Aglioti S. M. Cesari P. Romani M. Urgesi C. (2008). Action anticipation and motor resonance in elite basketball players. *Nat. Neurosci.* 11 1109–1116. 10.1038/nn.2182 19160510

[B2] Anelli F. Borghi A. M. Nicoletti R. (2012a). Grasping the pain: motor resonance with dangerous affordances. *Conscious. Cogn.* 21 1627–1639. 10.1016/j.concog.2012.09.001 23041720

[B3] Anelli F. Nicoletti R. Kalkan S. Sahin E. Borghi A. M. (2012b). “Human and robotics hands grasping danger,” in *Proceeding of the 2012 International Joint Conference on Neural Networks (IJCNN)* (Piscataway, NJ: IEEE), 1–8. 10.1109/IJCNN.2012.6252590

[B4] Anelli F. Ranzini M. Nicoletti R. Borghi A. M. (2013). Perceiving object dangerousness: an escape from pain? *Exp. Brain Res.* 228 457–466. 10.1007/s00221-013-3577-2 23743714

[B5] Aron A. Aron E. N. Smollan D. (1992). Inclusion of other in the self scale and the structure of interpersonal closeness. *J. Pers. Soc. Psychol.* 63 596–612. 10.1037/0022-3514.63.4.596

[B6] Avenanti A. Bueti D. Galati G. Aglioti S. M. (2005). Transcranial magnetic stimulation highlights the sensorimotor side of empathy for pain. *Nat. Neurosci.* 8 955–960. 10.1038/nn1481 15937484

[B7] Avenanti A. Minio-Paluello I. Sforza A. Aglioti S. M. (2009). Freezing or escaping? Opposite modulations of empathic reactivity to the pain of others. *Cortex* 45 1072–1077. 10.1016/j.cortex.2008.10.004 19100972

[B8] Avenanti A. Sirigu A. Aglioti S. M. (2010). Racial bias reduces empathic sensorimotor resonance with other-race pain. *Curr. Biol.* 20 1018–1022. 10.1016/j.cub.2010.03.071 20537539

[B9] Brambilla M. Sacchi S. Pagliaro S. Ellemers N. (2013). Morality and intergroup relations: threats to safety and group image predict the desire to interact with outgroup and ingroup members. *J. Exp. Soc. Psychol.* 49 811–821. 10.1016/j.jesp.2013.04.005

[B10] Brewer M. B. Gardner W. (1996). Who is this “We”? Levels of collective identity and self representations. *J. Pers. Soc. Psychol.* 71 83–93. 10.1037/0022-3514.71.1.83

[B11] Buccino G. Vogt S. Ritzl A. Fink G. R. Zilles K. Freund H. J. (2004). Neural circuits underlying imitation learning of hand actions: an event-related fMRI study. *Neuron* 42 323–334. 10.1016/S0896-6273(04)00181-3 15091346

[B12] Cameron J. E. (2004). A three-factor model of social identity. *Self Identity* 3 239–262. 10.1080/13576500444000047

[B13] Cappella J. N. (1997). Behavioral and judged coordination in adult informal social interactions: vocal and kinesic indicators. *J. Pers. Soc. Psychol.* 72 119–131. 10.1037/0022-3514.72.1.119

[B14] Chartrand T. L. Bargh J. A. (1999). The chameleon effect: the perception–behavior link and social interaction. *J. Pers. Soc. Psychol.* 76 893–910. 10.1037/0022-3514.76.6.893 10402679

[B15] Cheng Y. Lee P. L. Yang C. Y. Lin C. P. Hung D. Decety J. (2008). Gender differences in the mu rhythm of the human mirror-neuron system. *PLoS ONE* 3:e2113 10.1371/journal.pone.0002113 PMC2361218 18461176

[B16] Coello Y. Bourgeois J. Iachini T. (2012). Embodied perception of reachable space: how do we manage threatening objects? *Cogn. Process.* 13 131–135. 10.1007/s10339-012-0470-z 22806660

[B17] Cuddy A. J. Fiske S. T. Glick P. (2008). Warmth and competence as universal dimensions of social perception: the stereotype content model and the BIAS map. *Adv. Exp. Soc. Psychol.* 40 61–149. 10.1016/S0065-2601(07)00002-0

[B18] Decety J. Jackson P. L. (2004). The functional architecture of human empathy. *Behav. Cogn. Neurosci. Rev.* 3 71–100. 10.1177/1534582304267187 15537986

[B19] Désy M. C. Théoret H. (2007). Modulation of motor cortex excitability by physical similarity with an observed hand action. *PLoS ONE* 2:e971 10.1371/journal.pone.0000971 PMC1989142 17912350

[B20] Devine P. G. (1989). Stereotypes and prejudice: their automatic and controlled components. *J. Pers. Soc. Psychol.* 56 5–18. 10.1037/0022-3514.56.1.5

[B21] Devine P. G. Elliot A. J. (1995). Are racial stereotypes really fading? The Princeton trilogy revisited. *Pers. Soc. Psychol. Bull.* 21 1139–1150. 10.1177/01461672952111002

[B22] Dijksterhuis A. Bargh J. A. (2001). The perception-behavior expressway: automatic effects of social perception on social behavior. *Adv. Exp. Soc. Psychol.* 33 1–40. 10.1016/S0065-2601(01)80003-4

[B23] Dovidio J. F. Gaertner S. L. (2010). “Intergroup bias,” in *Handbook of Social Psychology*, 5th Edn Vol. 2 eds Fiske S. T. Gilbert D. Lindzey G. (New York, NY: John Wiley & Sons, Inc), 1084–1121.

[B24] Dolk T. Hommel B. Colzato L. S. Schütz-Bosbach S. Prinz W. Liepelt R. (2011). How “social” is the social Simon effect? *Front. Psychol.* 2:84 10.3389/fpsyg.2011.00084 PMC3110342 21687453

[B25] Fadiga L. Craighero L. Olivier E. (2005). Human motor cortex excitability during the perception of others’ action. *Curr. Opin. Neurobiol.* 15 213–218. 10.1016/j.conb.2005.03.013 15831405

[B26] Fadiga L. Fogassi L. Pavesi G. Rizzolatti G. (1995). Motor facilitation during action observation: a magnetic stimulation study. *J. Neurophysiol.* 73 2608–2611. 7666169 10.1152/jn.1995.73.6.2608

[B27] Fiske A. P. (1992). The four elementary forms of sociality: framework for a unified theory of social relations. *Psychol. Rev.* 99 689–723. 10.1037/0033-295X.99.4.689 1454904

[B28] Fiske S. T. Cuddy A. J. Glick P. (2007). Universal dimensions of social cognition: warmth and competence. *Trends Cogn. Sci.* 11 77–83. 10.1016/j.tics.2006.11.005 17188552

[B29] Fox E. Russo R. Dutton K. (2002). Attentional bias for threat: evidence for delayed disengagement from emotional faces. *Cogn. Emot.* 16 355–379. 10.1080/02699930143000527 18273395 PMC2241753

[B30] Freeman J. B. Ambady N. (2010). Mouse tracker: software for studying real-time mental processing using a computer mouse-tracking method. *Behav. Res. Methods* 42 226–241. 10.3758/BRM.42.1.226 20160302

[B31] Freeman J. B. Ambady N. Rule N. O. Johnson K. L. (2008). Will a category cue attract you? Motor output reveals dynamic competition across person construal. *J. Exp. Psychol. Gen.* 137 673 10.1037/a0013875 18999360

[B32] Freeman J. B. Ma Y. Han S. Ambady N. (2013). Influences of culture and visual context on real-time social categorization. *J. Exp. Soc. Psychol.* 49 206–210. 10.1016/j.jesp.2012.10.015 23355750 PMC3551594

[B33] Frischen A. Bayliss A. P. Tipper S. P. (2007). Gaze cueing of attention: visual attention, social cognition, and individual differences. *Psychol. Bull.* 133 694–724. 10.1037/0033-2909.133.4.694 17592962 PMC1950440

[B34] Frith C. D. (2007). The social brain? *Philos. Trans. R. Soc. Lond. B Biol. Sci.* 362 671–678. 10.1098/rstb.2006.2003 17255010 PMC1919402

[B35] Fourkas A. D. Avenanti A. Urgesi C. Aglioti S. M. (2006). Corticospinal facilitation during first and third person imagery. *Exp. Brain Res.* 168 143–151. 10.1007/s00221-005-0076-0 16044298

[B36] Gallese V. Fadiga L. Fogassi L. Rizzolatti G. (1996). Action recognition in the premotor cortex. *Brain* 119 593–609. 10.1093/brain/119.2.593 8800951

[B37] Gutsell J. N. Inzlicht M. (2010). Empathy constrained: prejudice predicts reduced mental simulation of actions during observation of outgroups. *J. Exp. Soc. Psychol.* 46 841–845. 10.1016/j.jesp.2010.03.011

[B38] Gutsell J. N. Inzlicht M. (2013). “Using EEG mu-suppression to explore group biases in motor resonance,” in *Neuroscience of Prejudice and Intergroup Relations*, eds Derks B. Scheepers D. Ellemers N. (Milton Park: Psychology Press), 279–297.

[B39] Hayes A. F. (2013). *Introduction to Mediation, Moderation, and Conditional Process Analysis: A Regression-Based Approach*. New York, NY: Guilford Press.

[B40] Hewstone M. Rubin M. Willis H. (2002). Intergroup bias. *Annu. Rev. Psychol.* 53 575–604. 10.1146/annurev.psych.53.100901.135109 11752497

[B41] Hilton J. L. von Hippel W. (1996). Stereotypes. *Annu. Rev. Psychol.* 47 237–271. 10.1146/annurev.psych.47.1.237 15012482

[B42] Hurley S. (2008). The shared circuits model (SCM): how control, mirroring, and simulation can enable imitation, deliberation, and mindreading. *Behav. Brain Sci.* 31 1–2; discussion 22–58. 10.1017/S0140525X07003123 18394222

[B43] Iacoboni M. (2005). Neural mechanisms of imitation. *Curr. Opin. Neurobiol.* 15 632–637. 10.1016/j.conb.2005.10.010 16271461

[B44] Iacoboni M. (2009). Imitation, empathy, and mirror neurons. *Annu. Rev. Psychol.* 60 653–670. 10.1146/annurev.psych.60.110707.163604 18793090

[B45] Iacoboni M. Woods R. P. Brass M. Bekkering H. Mazziotta J. C. Rizzolatti G. (1999). Cortical mechanisms of human imitation. *Science* 286 2526–2528. 10.1126/science.286.5449.2526 10617472

[B46] Kaplan J. T. Aziz-Zadeh L. Uddin L. Q. Iacoboni M. (2008). The self across the senses: an fMRI study of self-face and self-voice recognition. *Soc. Cogn. Affect. Neurosci.* 3 218–223. 10.1093/scan/nsn014 19015113 PMC2566765

[B47] Kilner J. M. Paulignan Y. Blakemore S. J. (2003). An interference effect of observed biological movement on action. *Curr. Biol.* 13 522–525. 10.1016/S0960-9822(03)00165-9 12646137

[B48] Knoblich G. Sebanz N. (2006). The social nature of perception and action. *Curr. Dir. Psychol. Sci.* 15 99–104. 10.1111/j.0963-7214.2006.00415.x

[B49] Liew S. L. Han S. Aziz-Zadeh L. (2011). Familiarity modulates mirror neuron and mentalizing regions during intention understanding. *Hum. Brain Mapp.* 32 1986–1997. 10.1002/hbm.21164 20882581 PMC6870503

[B50] Liuzza M. T. Candidi M. Sforza A. L. Aglioti S. M. (2015). Harm avoiders suppress motor resonance to observed immoral actions. *Soc. Cogn. Affect. Neurosci.* 10 72–77. 10.1093/scan/nsu025 24526183 PMC4994846

[B51] Losin E. A. R. Iacoboni M. Martin A. Cross K. A. Dapretto M. (2012). Race modulates neural activity during imitation. *Neuroimage* 59 3594–3603. 10.1016/j.neuroimage.2011.10.074 22062193 PMC3909702

[B52] Mange J. Chun W. Y. Sharvit K. Belanger J. J. (2012). Thinking about arabs and muslims makes americans shoot faster: effects of category accessibility on aggressive responses in a shooter paradigm. *Eur. J. Soc. Psychol.* 42 552–556. 10.1002/ejsp.1883

[B53] McConahay J. B. (1986). “Modern racism, ambivalence, and the modern racism scale,” in *Prejudice, Discrimination, and Racism*, eds Dovidio J. F. Gaertner S. L. (San Diego, CA: Academic Press), 91–125.

[B54] Miles L. K. Griffiths J. L. Richardson M. J. Macrae C. N. (2010). Too late to coordinate: contextual influences on behavioral synchrony. *Eur. J. Soc. Psychol.* 40 52–60. 10.1002/ejsp.721

[B55] Minio-Paluello I. Baron-Cohen S. Avenanti A. Walsh V. Aglioti S. M. (2009). Absence of embodied empathy during pain observation in Asperger syndrome. *Biol. Psychiatry* 65 55–62. 10.1016/j.biopsych.2008.08.006 18814863

[B56] Molnar-Szakacs I. Wu A. D. Robles F. J. Iacoboni M. (2007). Do you see what I mean? Corticospinal excitability during observation of culture-specific gestures. *PLoS ONE* 2:e626 10.1371/journal.pone.0000626 PMC1913205 17637842

[B57] Öhman A. Flykt A. Esteves F. (2001). Emotion drives attention: detecting the snake in the grass. *J. Exp. Psychol. Gen.* 130 466–478. 10.1037/0096-3445.130.3.466 11561921

[B58] Oswald D. L. (2005). Understanding Anti-Arab Reactions Post-9/11: the role of threats, social categories, and personal ideologies1. *J. Appl. Soc. Psychol.* 35 1775–1799. 10.1111/j.1559-1816.2005.tb02195.x

[B59] Payne B. K. (2001). Prejudice and perception: the role of automatic and controlled processes in misperceiving a weapon. *J. Personal. Soc. Psychol.* 81 181–192. 10.1037/0022-3514.81.2.181 11519925

[B60] Perani D. Fazio F. Borghese N. A. Tettamanti M. Ferrari S. Decety J. (2001). Different brain correlates for watching real and virtual hand actions. *Neuroimage* 14 749–758. 10.1006/nimg.2001.0872 11506547

[B61] Pettigrew T. F. (2008). Future directions for intergroup contact theory and research. *Int. J. Intercult. Relat.* 32 187–199. 10.1016/j.ijintrel.2007.12.002

[B62] Pettigrew T. F. Tropp L. R. (2008). How does intergroup contact reduce prejudice? Meta-analytic tests of three mediators. *Eur. J. Soc. Psychol.* 38 922–934. 10.1002/ejsp.504

[B63] Plant E. A. Devine P. G. (1998). Internal and external motivation to respond without prejudice. *J. Personal. Soc. Psychol.* 75 811–832. 10.1037/0022-3514.75.3.811 16055643

[B64] Posner M. I. (1978). *Chronometric Explorations of Mind.* Lawrence: Erlbaum.

[B65] Posner M. I. (1980). Orienting of attention. *Q. J. Exp. Psychol.* 32 3–25. 10.1080/00335558008248231 7367577

[B66] Press C. Cook J. Blakemore S. J. Kilner J. (2011). Dynamic modulation of human motor activity when observing actions. *J. Neurosci.* 31 2792–2800. 10.1523/JNEUROSCI.1595-10.2011 21414901 PMC3398132

[B67] Riek B. M. Mania E. W. Gaertner S. L. (2006). Intergroup threat and outgroup attitudes: a meta-analytic review. *Personal. Soc. Psychol. Rev.* 10 336–353. 10.1207/s15327957pspr1004_4 17201592

[B68] Righart R. De Gelder B. (2008). Recognition of facial expressions is influenced by emotional scene gist. *Cogn. Affect. Behav. Neurosci.* 8 264–272. 10.3758/CABN.8.3.264 18814463

[B69] Rizzolatti G. Craighero L. (2004). The mirror-neuron system. *Annu. Rev. Neurosci.* 27 169–192. 10.1146/annurev.neuro.27.070203.144230 15217330

[B70] Rizzolatti G. Fogassi L. Gallese V. (2001). Neurophysiological mechanisms underlying the understanding and imitation of action. *Nat. Rev. Neurosci.* 2 661–670. 10.1038/35090060 11533734

[B71] Rizzolatti G. Sinigaglia C. (2010). The functional role of the parieto-frontal mirror circuit: interpretations and misinterpretations. *Nat. Rev. Neurosci.* 11 264–274. 10.1038/nrn2805 20216547

[B72] Roelofs K. Hagenaars M. A. Stins J. (2010). Facing freeze social threat induces bodily freeze in humans. *Psychol. Sci.* 21 1575–1581. 10.1177/0956797610384746 20876881

[B73] Sacheli L. M. Christensen A. Giese M. A. Taubert N. Pavone E. F. Aglioti S. M. (2015). Prejudiced interactions: implicit racial bias reduces predictive simulation during joint action with an out-group avatar. *Sci. Rep.* 5:8507 10.1038/srep08507 PMC5389129 25687636

[B74] Sebanz N. Knoblich G. Prinz W. (2003). Representing others’ actions: just like one’s own? *Cognition* 88 B11–B21. 10.1016/S0010-0277(03)00043-X 12804818

[B75] Stephan W. G. Stephan C. W. (2000). “An integrated threat theory of prejudice,” in *Reducing Prejudice and Discrimination*, ed. Oskamp S. (Hillsdale, NJ: Erlbaum), 23–46.

[B76] Stephan W. G. Ybarra O. Bachman G. (1999). Prejudice toward immigrants. *J. Appl. Soc. Psychol.* 29 2221–2237. 10.1111/j.1559-1816.1999.tb00107.x

[B77] Trawalter S. Hoffman K. M. Waytz A. (2012). Racial bias in perceptions of others’ pain. *PLoS ONE* 7:e48546 10.1371/journal.pone.0048546 PMC3498378 23155390

[B78] Trawalter S. Todd A. R. Baird A. A. Richeson J. A. (2008). Attending to threat: race-based patterns of selective attention. *J. Exp. Soc. Psychol.* 44 1322–1327. 10.1016/j.jesp.2008.03.006 19727428 PMC2633407

[B79] Todorov A. Pakrashi M. Oosterhof N. N. (2009). Evaluating faces on trustworthiness after minimal time exposure. *Soc. Cogn.* 27 813–833. 10.1521/soco.2009.27.6.813

[B80] Tsai C. C. Brass M. (2007). Does the human motor system simulate Pinocchio’s actions? Coacting with a human hand versus a wooden hand in a dyadic interaction. *Psychol. Sci.* 18 1058–1062. 10.1111/j.1467-9280.2007.02025.x 18031412

[B81] Uddin L. Q. Molnar-Szakacs I. Zaidel E. Iacoboni M. (2006). rTMS to the right inferior parietal lobule disrupts self–other discrimination. *Soc. Cogn. Affect. Neurosci.* 1 65–71. 10.1093/scan/nsl003 17387382 PMC1832105

[B82] Urgesi C. Candidi M. Fabbro F. Romani M. Aglioti S. M. (2006). Motor facilitation during action observation: topographic mapping of the target muscle and influence of the onlooker’s posture. *Eur. J. Neurosci.* 23 2522–2530. 10.1111/j.1460-9568.2006.04772.x 16706859

[B83] Williams J. M. G. Mathews A. MacLeod C. (1996). The emotional Stroop task and psychopathology. *Psychol. Bull.* 120 3–24. 10.1037/0033-2909.120.1.3 8711015

[B84] Williams J. M. G. Watts F. N. MacLeod C. Mathews A. (1988). *Cognitive Psychology and Emotional Disorders.* Hoboken, NJ: John Wiley & Sons.

[B85] Wilson M. Knoblich G. (2005). The case for motor involvement in perceiving conspecifics. *Psychol. Bull.* 131 460–473. 10.1037/0033-2909.131.3.460 15869341

[B86] Wojciszke B. (2005). Morality and competence in person-and self-perception. *Eur. Rev. Soc. Psychol.* 16 155–188. 10.1080/10463280500229619

[B87] Wojciszke B. Bazinska R. Jaworski M. (1998). On the dominance of moral categories in impression formation. *Pers. Soc. Psychol. Bull.* 24 1251–1263. 10.1177/01461672982412001

